# Stent Graft-in-Stent Graft as a Rescue Technique for Endovascular Treatment of Giant Extracranial Internal Carotid Aneurysm

**DOI:** 10.1155/2016/2656421

**Published:** 2016-09-25

**Authors:** Adenauer Marinho de Oliveira Góes Junior, Salim Abdon Haber Jeha

**Affiliations:** ^1^Brazilian Society of Angiology and Vascular Surgery (SBACV), Belém, PA, Brazil; ^2^Federal University of Pará (UFPA), Belém, PA, Brazil; ^3^University Center of Pará State (CESUPA), Belém, PA, Brazil

## Abstract

Endovascular treatment of a giant extracranial internal carotid aneurysm by a stent graft implantation was unsuccessful due to a high flow leak directly through the stent graft's coating. The problem was solved deploying a second stent graft inside the previously implanted one resulting in complete exclusion of the aneurysmal sac and patent carotid lumen preservation. The review of the literature did not provide a case using this endovascular strategy. Follow-up for more than 12 months, using CT angiography, showed confirmed aneurysmal exclusion and carotid patency and no clinical complications have been detected.

## 1. Introduction

Extracranial carotid aneurysms are extremely rare, accounting for only 0.4–4% of all peripheral artery aneurysms and representing less than 2% of all carotid interventions [1–6].

In spite of the fact that most of these aneurysms are asymptomatic, previous studies reported a stroke prevalence of 50% and mortality of up to 70% when left untreated [4, 7].

Development of new devices and consolidation of interventional skills during the treatment of a sort of carotid conditions such as intracranial aneurysms, carotid blowout syndrome, spontaneous dissection, carotid stenosis, and pseudoaneurysms [1–14] have made endovascular techniques emerge as an attractive alternative for extracranial internal carotid aneurysms (EICA) and pseudoaneurysms (PA) [1].

Bare stents, coils, stent grafts, and occlusion balloons, among others, have been used to treat EICA and PA; these materials can be used alone or combined to each other in many fashions such as using as much as 4 bare stents deployed inside each other, bare stents overlapping, gradually tapering overlapping covered stents, and coil embolization through bare stents mesh [2, 3, 7, 8, 10–13, 15].

Technical success of endovascular procedures for EICA and PA is as high as 92.8%; endoleaks are the leading hospital complication, accounting for 8.1% of cases, and emergent surgical conversion is required in up to 7.7% of true aneurysms [1].

Because most vascular surgeons, even those at large centers, do not get enough personal experience operating on these cases [5], publications that bring new information about how to solve endovascular treatment complications without surgical conversion are helpful.

We describe results of an EICA repair using a stent graft intrastent graft strategy to correct an important endoleak and avoid open surgical conversion; patient has been followed up for more than 1 year.

According to the literature's review the use of a stent graft intrastent graft implanted at the carotid arteries has not been previously described.

## 2. Case Report

A 62-year-old woman presented a pulsatile mass on the right side of the neck with thrill and murmur. Except for being a smoker for a long time clinical history was unremarkable. CT angiography confirmed a saccular aneurysm at the middle third of the extracranial internal carotid; larger diameter was 3 cm; no significant stenosis was detected.

Endovascular treatment was planned. The procedure was carried out under general anesthesia and systemic anticoagulation (nonfractioned heparin). Right femoral access was used. Angiography of the aortic arch and selective catheterization of its branches excluded significant stenosis and intracranial aneurysms.

A vertebral catheter and a hydrophilic guidewire were placed at the distal right internal carotid; there materials were exchanged for an Amplatzer guidewire and an 8-French sheath to enhance support.

Under road map and using a 0.018-inch guidewire a 5 mm × 2.5 cm Viabahn stent graft (Gore®) was deployed sealing the aneurysmal neck; no protection device for distal embolization was used. Control angiography showed persistence of blood flows inside the aneurysm because of an intense leak directly through the stent graft coating. After 15 minutes a control angiography showed no diminishment of the leak intensity (a video showing the leak is available as Supplementary Material, available online at http://dx.doi.org/10.1155/2016/2656421).

**Figure 1:**
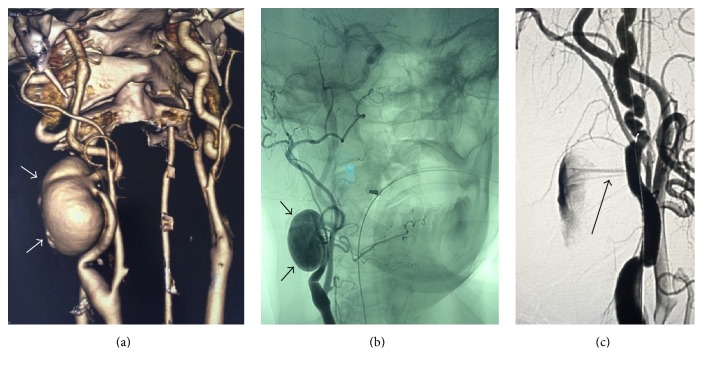
(a) Preoperative CT, the arrow points to the aneurysm; (b) intraoperative angiography, the arrow points to the aneurysm; (c) intraoperative angiography after first stent graft deployment, the arrow points to the leak.

The only stent graft available was a Viabahn 5 mm × 5 cm. A second 0.018-inch guidewire was placed in parallel to first guidewire in order to enhance support for the second stent graft navigation. Under road map this second stent was deployed inside the previously implanted one.  Figure 1 demonstrates the preoperative CT and intraoperative angiographies.

Control angiography showed complete aneurysm exclusion, patency of the carotid artery, and no signs of distal embolization.

After the procedure patient received 200 mg of aspirin and 300 mg of clopidogrel.

Patient remained in the ICU for 24 hours and was discharged after 48 hours.

Aspirin (100 mg/day), clopidogrel (75 mg/day), and atorvastatin (40 mg/day) were prescribed for continuous use.

**Figure 2:**
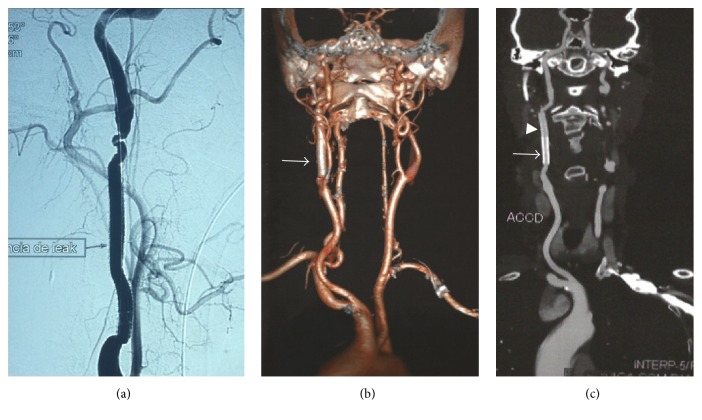
(a) Intraoperative angiography after second stent graft deployment, patent carotid, and leak absence; (b) one-year CT control, arrow points to the stent grafts, no signs of stents occlusion; (c) one-year CT control, arrow points to the first deployed stent grafts and arrow head points to the second stent graft.

Patient has been followed up for 18 months; she gave up smoking and the neck bulging disappeared four months after surgery. Duplex scan after 3 months and angio-CT after 6 months and 12 months showed persistence of aneurysm exclusion and carotid artery patency with minimum signs of intimal hyperplasia.  Figure 2 shows the immediate angiographic control after the second stent deployment and the control after one year by CT.

## 3. Discussion

A search on PubMed for “carotid aneurysm” or “carotid peseudoaneurysm” and “stent” on title/abstracts showed 75 articles published between 1997 and 2016. These terms were chosen because they have been previously used by other authors [1, 9]. All titles were reviewed; those which contained “stent-graft”, “stent graft”, “overlapping stent”, “Viabahn”, and “covered stent” had their abstract read. To the best of our knowledge this is the first time the use of a stent graft-in-stent graft has been reported in the carotid territory.

In 1808 Sir Astley Cooper ligated the common carotid artery during the first surgery for an extracranial carotid aneurysm [1, 3, 6].

Based on the CEA location Attigah classified and proposed surgical strategies [6]; the aneurysm in this case would be type I (isolated internal carotid aneurysm above the carotid bulb), treatable by both open and endovascular techniques [6].

Open surgery carries about 2.2% postoperative mortality but is criticized for its high cranial nerve injury incidence, from 2.2 up to 44% [1]. Open surgery was considered a second option for this case because the upper limit of the aneurysm was behind the mandible, increasing the risks during dissection.

Endovascular methods have gained popularity as interventionists had access to new endovascular devices [2, 13]. Because true extracranial carotid aneurysms (ECA) are rare [4–6], much of the endovascular experience applied to their treatment was accumulated by treating other challenging carotid conditions such as carotid stenosis, dissection, carotid blowout syndrome, intracranial aneurysms, and traumatic pseudoaneurysms [1, 8, 11–15]. Endovascular procedures for ECA are successful in 92.8% and have 4.1% in-hospital mortality and stroke and cranial nerve injury occur in 1.8 and 0.5%, respectively [1].

Embolic protection devices were not used since filter guidewires do not offer the necessary stiffness to covered stent navigation and also because angiography showed no evidence of significant atherosclerosis or thrombus.

Most authors used aspirin and clopidogrel for antiaggregation [9, 11, 12, 15], but dosage, the option to start the drugs before or at the day the procedure was performed, and for how long dugs were used after the procedure diverged. Clopidogrel prescription varied from 3 weeks to 6 months [9, 11, 15]; most of them used aspirin indefinitely after the procedure (250 mg to 325 mg/day) [9, 11, 15]. Because of the lack of experience with stent graft-in-stent graft implantation at the carotid territory, aspirin, clopidogrel, and atorvastatin were maintained.

Accurate sizing of covered stents relative to the vessel landing zones is important in attaining favorable results. The internal carotid artery typically measures 5.11 mm in men and 4.66 mm in women [8], so the 5 mm stent grafts would fit most of women internal carotids.

Even if a single stent graft was implanted, as planned, some antiaggregation would be needed for months due to the known thrombogenicity of these stents [10, 12, 15] but this could lead to endoleaks maintenance, so the immediate exclusion of the aneurysmal sac was a priority.

Techniques requiring direct aneurysm puncture were not considered because it would result in persistent neck bulging if coils were used to embolize such a big aneurysm and thrombin injection would require the undesirable aneurysm compression. So a second stent graft deployment was chosen; if a covered stent with the same length (2.5 cm) of the first one was available it would be preferable, but since it was not possible a stent with the same diameter but with a 5.0 cm was deployed inside the first one.

Obvious concern regards intimal hyperplasia and thrombosis, but previous reports of overlapping bare stents use demonstrated that intimal hyperplasia is typically mild and not hemodynamically significant and does not increase occlusion or other delayed complication [11] and also that maximum endothelial growth occurred in a vessel segment covered by a single stent, not in the area of stent overlap, suggesting the inner stent does not promote significant additional injury to the vessel wall [15]. In this case there were no radiological or clinical signs of significant complications more than 18 months after the endovascular treatment.

## 4. Conclusion

Because ECA is rarely diagnosed most surgeons/interventional radiologists do not accumulate large treatment experience. In selected cases the stent graft-in-stent graft strategy can avoid surgical conversion when dealing with endovascular procedure's complication not only for ECA but other more common carotid territory conditions.

## Supplementary Material

The video shows a persistent high flow leak through the stent graft mash.
